# Bacterial Translocation As the Origin of Gram-Negative Rods Bloodstream Infection Among Older Patients in Rural Hospitals: A Cross-Sectional Study

**DOI:** 10.7759/cureus.50706

**Published:** 2023-12-18

**Authors:** Ryuichi Ohta, Chiaki Sano

**Affiliations:** 1 Community Care, Unnan City Hospital, Unnan, JPN; 2 Community Medicine, Shimane University Faculty of Medicine, Izumo, JPN

**Keywords:** family medicine, bloodstream infections, gram-negative bacteremia, escherichia coli, rural healthcare, bacterial translocation, elderly patients

## Abstract

Introduction

Bloodstream infections caused by Gram-negative rods are a pressing concern for the aging global population, particularly in rural settings. This study investigates the prevalence and entry pathways of Gram-negative rod bloodstream infections in elderly patients at a rural Japanese hospital, aiming to clarify the frequency and associated factors of straightforward entry and bacterial translocation.

Method

In this cross-sectional study, we analyzed electronic medical records of patients over 18 years of age with symptomatic Gram-negative rod bloodstream infections at Unnan City Hospital, Japan, from September 2021 to August 2023. We used multivariate logistic regression to assess factors of age, sex, body mass index, care dependency, and comorbidities.

Results

Among the participants who met the inclusion criteria, significant differences were observed in age, sex, inpatient status, and prevalence of conditions like respiratory diseases and cancer between the straightforward entry and bacterial translocation groups. *Escherichia coli* was the most common pathogen identified.

Conclusion

The study emphasizes the need for tailored medical approaches for elderly patients with bloodstream infections, considering their unique health profiles and risks. It highlights the importance of age, inpatient status, and cancer in determining infection risks, pointing to areas for further research to enhance infection management and healthcare outcomes in older populations.

## Introduction

Bloodstream infections, particularly those caused by Gram-negative rods, are increasingly significant health threats for the aging global population [1]. These infections are especially critical for older patients, presenting heightened risks and potential fatalities [2]. This demographic's vulnerability underscores the need for an in-depth study of Gram-negative rod bloodstream infections in the elderly [3]. Such research is crucial for developing optimized treatment strategies and preventive measures to protect older individuals from recurring infections.

Despite growing concerns about these infections, the extent of their prevalence among older patients, especially in rural areas, remains unclear [4]. Understanding this prevalence is vital, academically, and as a basis for effective healthcare interventions for this at-risk group [5].

A key aspect of addressing these infections is understanding how pathogens enter the bloodstream [6]. Identifying these entry points can revolutionize clinical approaches, enhancing diagnosis and treatment for older patients and reducing recurrent infections. Bacterial translocation is a significant factor in these infections [7,8], commonly occurring in the gastrointestinal tract [9]. Conditions that suppress the immune system can also lower defenses in the gastrointestinal and respiratory tracts, leading to bloodstream infections [10]. However, bacterial translocation frequency in this context has yet to be fully understood.

This study aimed to thoroughly investigate the prevalence, entry pathways, and risk factors associated with Gram-negative rod bloodstream infections, focusing on bacterial translocation in the rural elderly population. We aimed to provide critical insights for the academic community and enhance clinical care for this vulnerable demographic in rural settings.

## Materials and methods

This cross-sectional study aimed to determine the frequency, demographics, and associated factors of Gram-negative rod bloodstream infections in rural hospitals. We analyzed electronic medical record data, employing multivariate logistic regression with common infection sites (urinary tract, gastrointestinal, hepatobiliary) as the dependent variable. Covariates included age, sex, body mass index, dependency according to Japanese long-term health insurance, comorbidities, and diagnosis.

Setting

In 2022, Unnan City's population was 35,738, with 40.27% aged 65 years or older. The city's sole public hospital had 281 beds, distributed across acute, general, rehabilitation, and chronic care units. The hospital's internal medicine patients were managed collaboratively by the Department of Family Medicine [11].

Participants

Participants were patients at Unnan City Hospital, Japan, over 18 years of age, presenting with local and systemic infectious symptoms, and a positive blood culture for Gram-negative rods. Exclusions were patients without infection symptoms, those with upper respiratory or skin infections, and those without a blood culture [12,13]. This study focused on bloodstream infections originating from urinary, gastrointestinal, and hepatobiliary tracts or bacterial translocation. Patient data from September 2021 to August 2023 were collected.

Data collection

The dependent variable was the presence of bloodstream infections from the urinary, gastrointestinal, and hepatobiliary tracts. Infections were diagnosed based on physician assessment and culture concordance between blood and other bodily fluids [3]. Bacterial translocation was identified as Gram-negative rod detection in blood culture without organ-specific symptoms [9]. Risk factors were based on previous studies, with data extracted from electronic records [12,13]. Covariates included age, sex, BMI, care dependency, and comorbidities.

Analysis

Continuous variables were tested for normality. The Student's t-test and Mann-Whitney U test analyzed parametric and nonparametric data, respectively. Categorical data were analyzed using the chi-squared test. Variables for logistic regression included sex, care dependency, and infection entry type (straightforward or bacterial translocation). The regression model included variables significant in univariate models [12,13]. All statistical analyses were performed using EZR (version 1.23) (Saitama, Japan: Saitama Medical Center, Jichi Medical University) and a graphical user interface for R (Vienna, Austria: The R Foundation) [14]. Statistical significance was set at p<0.05.

Ethical consideration

This study was approved by the Unnan City Hospital Clinical Ethics Committee (#20230025). Measures were taken to ensure patient confidentiality and data security in compliance with ethical guidelines.

## Results

**Figure 1:**
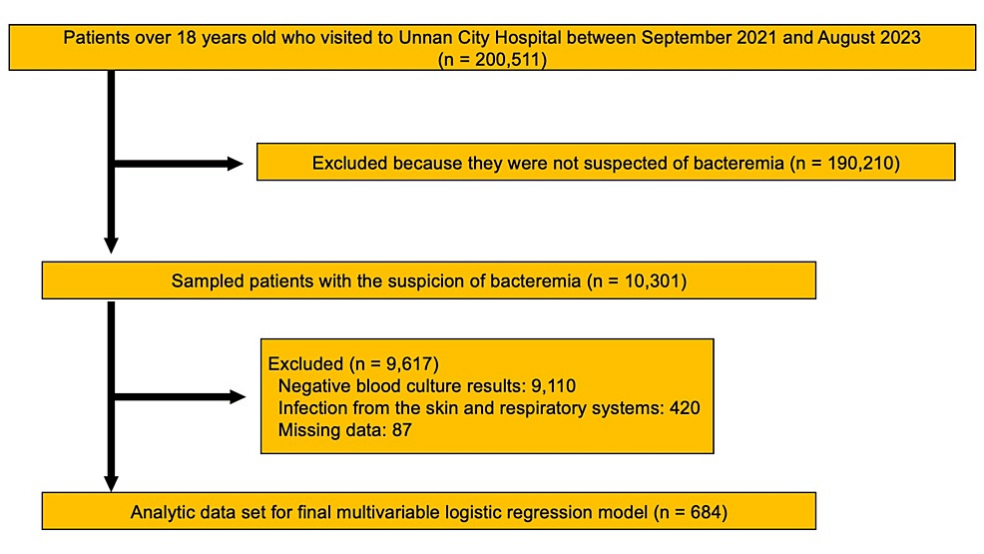
Selection process flowchart of the study participant.

Figure 1 shows the flowchart of the study participant selection process. Between September 2021 and August 2023, 200,511 patients visited the hospital with acute symptoms. Based on medical records, blood culture tests were performed on 10,301 patients with suspected bacteremia. A total of 9,617 patients were excluded based on the exclusion criteria. In total, 684 participants were included in this study.

Participant demographics

Our study involved 684 participants categorized into two groups as follows: straightforward entry (408 participants) and bacterial translocation (276 participants). The mean age was 84.68 years, with a standard deviation (SD) of 11.23. Notably, there were significant differences in age, sex distribution, dependent status, inpatient status, prevalence of respiratory diseases, and cancer between the two groups. In the straightforward entry group, a lower percentage of participants had respiratory diseases (1.5%) and were inpatients (26.5%) compared to the bacterial translocation group, where the corresponding percentages were 5.8% and 48.9%. Cancer incidence was also notably higher in the straightforward entry group (48.5%) compared to the bacterial translocation group (36.2%) (Table 1).

**Table 1:** The demographics of the participants. CKD: chronic kidney diseases; SD: standard deviation Respiratory diseases consist of asthma and chronic obstructive pulmonary diseases.

Factor	Overall	Origin		p-Value
		Straightforward entry	Bacterial translocation	
n	684	408	276	
Age, mean (SD)	84.68 (11.23)	83.78 (11.70)	86.01 (10.38)	0.011
Sex (%)	309 (45.2)	155 (38.0)	154 (55.8)	<0.001
Hight, mean (SD)	152.92 (9.98)	152.31 (10.00)	153.83 (9.90)	0.051
Weight, mean (SD)	45.85 (10.89)	45.59 (10.46)	46.23 (11.49)	0.456
BMI, mean (SD)	19.49 (3.56)	19.57 (3.55)	19.36 (3.57)	0.46
Dependent (%)	165 (24.1)	82 (20.1)	83 (30.1)	0.003
Inpatient (%)	243 (35.5)	108 (26.5)	135 (48.9)	<0.001
Respiratory diseases (%)	22 (3.2)	6 (1.5)	16 (5.8)	0.003
Brain stroke (%)	91 (13.3)	47 (11.5)	44 (15.9)	0.108
Cancer (%)	298 (43.6)	198 (48.5)	100 (36.2)	0.002
CKD (%)	20 (2.9)	12 (2.9)	8 (2.9)	1
Dementia (%)	49 (7.2)	23 (5.6)	26 (9.4)	0.07
Diabetes (%)	163 (23.8)	79 (19.4)	84 (30.4)	0.001
Heart failure (%)	222 (32.5)	111 (27.2)	111 (40.2)	<0.001

Organisms identified in blood cultures

Blood culture tests revealed various organisms, with Escherichia coli being the most common in both groups (54.7% in straightforward entry, 44.9% in bacterial translocation). However, certain organisms like Acinetobacter species, *Citrobacter freundii* complex, and *Clostridium perfringens* were exclusively found in the bacterial translocation group (Table 2).

**Table 2:** Concrete bacteria from blood culture tests. ESBL: extended spectrum beta-lactamase

Organism, n (%)	Origin	
	Straightforward entry	Bacterial translocation
	408	276
Acinetobacter species	0 (0.0)	4 (1.4)
*Aeromonas hydrophila* group	2 (0.5)	0 (0.0)
Bacteroides fragilis	0 (0.0)	1 (0.4)
Bacteroides ovatus	0 (0.0)	1 (0.4)
Citrobacter braakii	0 (0.0)	1 (0.4)
*Citrobacter freundii* complex	0 (0.0)	4 (1.4)
Citrobacter koseri	0 (0.0)	2 (0.7)
Clostridium innocuum	0 (0.0)	1 (0.4)
Clostridium perfringens	0 (0.0)	8 (2.9)
Clostridium ramosum	0 (0.0)	2 (0.7)
Enterobacter aerogenes	13 (3.2)	0 (0.0)
Enterobacter agglomerans	0 (0.0)	2 (0.7)
Enterobacter asburiae	0 (0.0)	2 (0.7)
Enterobacter cloacae	3 (0.7)	10 (3.6)
Escherichia coli	223 (54.7)	124 (44.9)
*E. coli* ESBL	43 (10.5)	18 (6.5)
Fusobacterium necrophorum	0 (0.0)	6 (2.2)
Haemophilus influenzae	0 (0.0)	2 (0.7)
Klebsiella oxytoca	11 (2.7)	10 (3.6)
Klebsiella ozaenae	0 (0.0)	1 (0.4)
Klebsiella pneumoniae	70 (17.2)	44 (15.9)
Listeria monocytogenes	1 (0.2)	0 (0.0)
Morganella morganii	16 (3.9)	0 (0.0)
Proteus mirabilis	12 (2.9)	9 (3.3)
Proteus vulgaris	4 (1.0)	2 (0.7)
Pseudomonas aeruginosa	4 (1.0)	11 (4.0)
Pseudomonas fluorescens/putida	0 (0.0)	1 (0.4)
Raoultella ornithinolytica	2 (0.5)	0 (0.0)
Rothia mucilaginosa	0 (0.0)	4 (1.4)
Serratia liquefaciens	0 (0.0)	2 (0.7)
Serratia marcescens	4 (1.0)	2 (0.7)
Yersinia frederiksenii	0 (0.0)	2 (0.7)

The result of the multivariate logistic regression model with the dependent variable of Gram-negative bloodstream infection from straightforward entry

Our multivariate logistic regression analysis indicated several significant factors. Age and inpatient status were negatively correlated with the presence of bloodstream infections of Gram-negative rods from straightforward entry (odds ratio {OR}: 0.97, 95% confidence interval {CI}: 0.96-0.99; and OR: 0.44, 95% CI: 0.30-0.63, respectively), positively correlated with the presence of bacterial translocation. At the same time, cancer significantly increased the odds of the presence of bloodstream infections of Gram-negative rods from straightforward entry (odds ratio: 2.12, 95% confidence interval {CI}: 1.46-3.08). Respiratory diseases and female sex were associated with decreased odds of the presence of straightforward infections of Gram-negative rods from bacterial translocation (OR: 0.14, 95% CI: 0.05-0.42; and OR: 0.31, 95% CI: 0.21-0.47, respectively), positively correlated with the presence of bacterial translocation (Table 3).

**Table 3:** The result of the multivariate logistic regression model with the dependent variable of Gram-negative bloodstream infection from straightforward entry. CKD: chronic kidney diseases Respiratory diseases consist of asthma and chronic obstructive pulmonary diseases.

Factor	Odds ratio	95% CI	p-Value
Age	0.97	0.96-0.99	0.0049
Sex (female)	0.31	0.21-0.47	<0.001
BMI	1.01	0.97-1.06	0.59
Dependent	1.21	0.78-1.86	0.39
Inpatient	0.44	0.30-0.63	<0.001
Respiratory diseases	0.14	0.05-0.42	<0.001
Cancer	2.12	1.46-3.08	<0.001
Brain stroke	0.83	0.50-1.37	0.46
CKD	2.78	0.95-8.13	0.061
Dementia	0.67	0.36-1.25	0.21
Diabetes	1.03	0.67-1.58	0.9
Heart failure	0.7	0.47-1.05	0.088

## Discussion

The current study offers a comprehensive analysis of the demographics, blood culture organisms, and logistic regression insights involving 684 elderly participants with varying health conditions, specifically straightforward entry and bacterial translocation. This investigation reveals that aging, female sex, inpatient conditions, and respiratory diseases positively correlate with bacterial translocation. In contrast, cancer is more commonly associated with straightforward entry of Gram-negative rod bloodstream infections.

In rural contexts, older patients exhibit a higher risk of Gram-negative rod bloodstream infections from bacterial translocation than younger counterparts. Our study corroborates that higher age positively correlates with bacterial translocation, aligning with findings from previous literature [15,16]. The immunosuppressive state and increased vulnerability to infections and treatments in older patients, coupled with multimorbidity and polypharmacy, alter their gut bacterial flora [17]. These changes potentially create conducive conditions for bacterial translocation [18]. Additionally, the frailty prevalent in older individuals impacts their gut conditions [19]. Oral and gastrointestinal frailty can modify their eating habits, subsequently affecting their gut flora, which may weaken the gut barrier and facilitate bacterial translocation [20].

Contrary to prior research, our study suggests an increased risk of Gram-negative rod bloodstream infection from bacterial translocation in females compared to males in rural settings [21,22]. Previous studies indicated that females are more susceptible to urinary tract infections due to anatomical differences, a common precursor to bloodstream infections [21,22]. The heightened risk of bacterial translocation in females observed in our study might be explained by variations in healthcare utilization patterns [23,24]. Females potentially seeking medical care more promptly for urinary tract infections could reduce the progression of these infections to bloodstream infections. Gender differences in help-seeking behaviors among older patients may influence the presentation of diseases and, consequently, the outcomes observed in this study [24].

The study's positive correlation between inpatient status and bloodstream infections from bacterial translocation challenges some traditional assumptions. It suggests that inpatients might be at a heightened risk for urinary tract infections due to various factors, including aggressive medical interventions and increased exposure to nosocomial pathogens [25]. The controlled environment of inpatients, including diet and hygiene managed by healthcare professionals, may reduce the risk of urinary and gastrointestinal tract infections [26]. However, hospital admission and treatments' psychological and physical stresses can weaken systemic immunity, leading to various hospital-acquired infections, including bacterial translocation.

The association of respiratory diseases with an increased likelihood of bacterial translocation is noteworthy, possibly reflecting underlying immunological differences [27]. The higher prevalence of respiratory diseases in the bacterial translocation group suggests a link between these conditions and the risk of bacterial translocation [28]. This aligns with previous findings that respiratory conditions and hospitalization elevate the vulnerability to bacterial infections due to compromised immunity and exposure to hospital-acquired pathogens [28]. This article shows that bacterial translocation is predominantly associated with nosocomial organisms such as Acinetobacter, Citrobacter, Pseudomonas, and Clostridium, except for *E. coli *and Klebsiella. This study thus highlights the potential for bacterial translocation originating from the respiratory system in older patients with chronic respiratory diseases.

In the context of cancer, our study highlights a significant increase in the odds of Gram-negative rod bloodstream infections originating from straightforward entry pathways. This observation further examines the interplay between cancer and susceptibility to specific types of infections. The immunosuppressive nature of cancer or its treatments like chemotherapy, which often weaken the body's defense mechanisms, may contribute to this increased risk [27]. While systematic immunosuppression due to cancer elevates the risk of various infections, our findings specifically indicate a higher likelihood of straightforward entry bacteremia as a prevalent infection than bacteremia resulting from bacterial translocation [29]. This underscores the critical need for enhanced infection control measures in oncology settings.

*E. coli* was the most frequently identified organism in blood cultures for both groups, in agreement with existing literature that recognizes it as a common cause of bloodstream infections in the elderly [30]. In the bacterial translocation group, the exclusive presence of specific organisms, such as Acinetobacter species and *Clostridium perfringens*, highlights distinct microbial profiles associated with different health conditions influenced by age, treatment-related immunosuppression, and hospital-acquired infections. These insights underscore the importance of accurate and timely organism identification for effective treatment.

This study, however, is not without limitations. The high average age of the study participants may limit the findings' applicability to younger demographics. The retrospective design precludes the establishment of causal relationships between health conditions and infection types. Moreover, the potential influence of unconsidered confounding factors, such as socioeconomic status and lifestyle choices, on the results cannot be discounted. Finally, the reliance on blood culture results may not capture subclinical infections or infections caused by fastidious organisms and previously partial usage of antibiotics, which could bias the understanding of the microbial landscape in these patient groups.

## Conclusions

This study offers vital insights into the dynamics of Gram-negative rod bloodstream infections among the elderly in a rural Japanese setting, drawing on data from 684 patients. Key findings include the significant influence of age, inpatient status, and cancer on infection risks. Older age and inpatient conditions were notably associated with bacterial translocation, a critical infection entry pathway. Conversely, cancer emerged as a significant factor for straightforward entry infections, highlighting the need for heightened infection control in oncology settings. The study identified *E. coli *as the predominant organism in bloodstream infections, underscoring its role in this patient demographic. Importantly, these results emphasize the necessity of personalized medical approaches for elderly patients, considering their unique health profiles and associated infection risks. Future research should focus on extending these findings to enhance infection management strategies, thereby improving healthcare outcomes for the elderly, especially in rural contexts. This study's insights are pivotal for developing targeted interventions and preventive measures to combat bloodstream infections in vulnerable elderly populations.
